# Nuclear Factor Erythroid-2-Related Factor 2 Signaling in the Neuropathophysiology of Inherited Metabolic Disorders

**DOI:** 10.3389/fncel.2021.785057

**Published:** 2021-11-26

**Authors:** Bianca Seminotti, Mateus Grings, Paolo Tucci, Guilhian Leipnitz, Luciano Saso

**Affiliations:** ^1^Postgraduate Program in Biological Sciences: Biochemistry, Department of Biochemistry, Institute of Basic Health Sciences, Universidade Federal do Rio Grande do Sul, Porto Alegre, Brazil; ^2^Department of Clinical and Experimental Medicine, University of Foggia, Foggia, Italy; ^3^Department of Biochemistry, Institute of Basic Health Sciences, Federal University of Rio Grande do Sul, Porto Alegre, Brazil; ^4^Postgraduate Program in Biological Sciences: Physiology, Institute of Basic Health Sciences, Universidade Federal do Rio Grande do Sul, Porto Alegre, Brazil; ^5^Department of Physiology and Pharmacology “Vittorio Erspamer”, Sapienza University of Rome, Rome, Italy

**Keywords:** Nrf2 signaling, antioxidant defenses, inherited metabolic disorders, neurometabolism, neurodegeneration

## Abstract

Inherited metabolic disorders (IMDs) are rare genetic conditions that affect multiple organs, predominantly the central nervous system. Since treatment for a large number of IMDs is limited, there is an urgent need to find novel therapeutical targets. Nuclear factor erythroid-2-related factor 2 (Nrf2) is a transcription factor that has a key role in controlling the intracellular redox environment by regulating the expression of antioxidant enzymes and several important genes related to redox homeostasis. Considering that oxidative stress along with antioxidant system alterations is a mechanism involved in the neuropathophysiology of many IMDs, this review focuses on the current knowledge about Nrf2 signaling dysregulation observed in this group of disorders characterized by neurological dysfunction. We review here Nrf2 signaling alterations observed in X-linked adrenoleukodystrophy, glutaric acidemia type I, hyperhomocysteinemia, and Friedreich’s ataxia. Additionally, beneficial effects of different Nrf2 activators are shown, identifying a promising target for treatment of patients with these disorders. We expect that this article stimulates research into the investigation of Nrf2 pathway involvement in IMDs and the use of potential pharmacological modulators of this transcription factor to counteract oxidative stress and exert neuroprotection.

## Inherited Metabolic Disorders and Neurological Dysfunction

The central nervous system (CNS) is a complex structure formed by distinct cell types organized in interacting substructures, whose development is dependent on the interactions between several single genes and non-genetic factors. Thus, CNS is highly susceptible to disturbances caused by genetic mutations that may be associated to external damaging factors (e.g., infection or vaccination), resulting in a broad range of disorders, including the so-called inherited metabolic disorders (IMDs) (Rose et al., 2020; Schiller et al., 2020).

Inherited metabolic disorders are a heterogeneous group of disorders caused by mutations that may affect thousands of molecules and proteins of human metabolism, mostly enzymes, cofactors, receptors and transporters, disrupting metabolic networks that underlie development and homeostasis. More than 1,100 different IMDs have been identified so far (Ferreira et al., 2019, 2021) and most of them involve the nervous system (Mankad et al., 2018). IMDs can lead to disruption of enzyme activity, cellular transport, or energy production, altering the metabolism of small or complex molecules and therefore causing their accumulation in tissues and biological fluids (Saudubray and Garcia-Cazorla, 2018b). The accumulation of these molecules, the reduced ability to synthesize intermediates or the defects in energy supply can severely interfere with the complex and finely tuned process of early brain development (Schiller et al., 2020). Thus, many IMDs are prevalent as diseases of the nervous system, being called neurometabolic diseases (García-Cazorla and Saudubray, 2018; Karimzadeh et al., 2020).

A simplified classification for IMDs affecting neurodevelopment has been proposed, based on the diagnostic approach, pathophysiology and size of accumulating molecules (small and simple or large and complex) in each disorder (Saudubray and Garcia-Cazorla, 2018a). Three large categories have been determined: disorders of small molecules, energy-related defects and complex molecule defects. It should be noted that the accumulating metabolites, independent of their size, may behave in the brain as signaling molecules, structural components and fuels so that alterations in their levels may severely damage neural cells (García-Cazorla and Saudubray, 2018).

The onset of symptoms in neurometabolic diseases can occur from neonatal period to adult life. A number of them may be present even after a period of normal growth and development. The estimated general prevalence is 1 in 1,000 live births (Karimzadeh et al., 2020). Clinical manifestations are diverse and may include seizures, global developmental delay, neuropathy, hypomyelination, microcephaly, and motor alterations (Mankad et al., 2018; Saudubray and Garcia-Cazorla, 2018a; Karimzadeh et al., 2020). Metabolic acidosis, hyperammonemia and lactic acidemia are common laboratory findings. The latter is mainly observed in disorders of small molecules and energy-related defects (García-Cazorla and Saudubray, 2018).

Many neurometabolic diseases have limited treatment so that early diagnosis is crucial to prevent devastating and long-term complications (Karimzadeh et al., 2020). In this scenario, several studies have aimed to characterize changes at molecular and structural levels, as well as alterations in redox homeostasis and energy metabolism in these diseases as an effort to discover novel therapeutical targets for these disorders (Swerdlow, 2016; García-Cazorla and Saudubray, 2018).

## Oxidative Stress in the Neuropathophysiology of Inherited Metabolic Disorders

A great amount of evidence shows that oxidative stress contributes to the pathogenesis of the neurological dysfunction in various IMDs (Mc Guire et al., 2009; Ribas et al., 2014; Wajner et al., 2020). Biomarkers of lipid, protein and DNA oxidative damage and altered antioxidant defenses, have been demonstrated in *in vitro* and *in vivo* disease models, as well as in cells of patients affected by these disorders (Mc Guire et al., 2009; Halliwell and Gutteridge, 2015; Faverzani et al., 2017; Parmeggiani and Vargas, 2018; Richard et al., 2018; Wajner et al., 2019, 2020).

As aforementioned, it is important to highlight that the brain has a high rate of oxidative metabolism coupled to reactive oxygen species (ROS) production, high amount of iron and greater peroxidation potential due to its elevated content of polyunsaturated fatty acids, and considerable low levels of antioxidant defenses, making this tissue highly vulnerable to oxidative stress (Halliwell, 2006; Halliwell and Gutteridge, 2015). In this scenario, it has been widely demonstrated that the metabolites accumulated in some inherited neurometabolic disorders, including amino acids, organic acids and acylcarnitines, act as neurotoxins and cause mitochondrial dysfunction leading to augmented generation of reactive species. Many of these neurotoxins disrupt mitochondrial homeostasis by causing disturbances in electron transport chain, which lead to increased electron leakage and a consequent overproduction of ROS (Wajner et al., 2004, 2019, 2020; Berger et al., 2014; Ribas et al., 2014; Leipnitz et al., 2015; Wyse et al., 2021). Therefore, the quantification of oxidative stress biomarkers may provide an approach for monitoring of redox status in individuals affected by IMDs and elucidation of a possible role of mitochondrial dysfunction in these disorders (Mc Guire et al., 2009; Leipnitz et al., 2015).

Treatment for many IMDs is limited and usually involves dietary restriction, supplementation of metabolic deficiencies and controlling of the main symptoms (Ribas et al., 2014; Saudubray and Garcia-Cazorla, 2018b; Kripps et al., 2021). Thus, in the last two decades, there has been intense research focusing on the development of new drugs aiming to target specific neural processes that have been shown to be disturbed in these disorders (Mazzola et al., 2013; Ribas et al., 2014; El-Hattab et al., 2017; Wajner, 2019). In this regard, antioxidant molecules were demonstrated to prevent not only oxidative stress, but also neurocognitive deficits observed in animal models of inherited neurometabolic diseases (Ribas et al., 2014; Leipnitz et al., 2015). Nevertheless, there is no consensus about the beneficial effects of antioxidants since this approach seems to be promising in preventing or dealing with a non-established process instead of reverting an installed damage.

Based on these observations, the modulation of nuclear factor erythroid-2-related factor 2 (Nrf2) signaling pathway has been studied as a therapeutical target for diseases related to oxidative stress and inflammation. Nrf2 modulators, mainly activators, have been already demonstrated to elicit beneficial effects in models of different human pathologies, such as cancer, autoimmune diseases, liver, kidney, and lung diseases, as well as common neurodegenerative disorders (Adelusi et al., 2020; Panieri et al., 2020; Scuderi et al., 2020; Sharma et al., 2020; Kim, 2021; Kourakis et al., 2021; Sezgin-Bayindir et al., 2021; Telkoparan-Akillilar et al., 2021; Zhao et al., 2021). Of note, some Nrf2 modulators are being currently evaluated in clinical trials for different disorders, such as cancer, neurodegenerative disorders and autoimmune diseases (Chartoumpekis et al., 2020; Panieri et al., 2020; Scuderi et al., 2020). In the following topics we will discuss recent evidence showing that Nrf2 pathway is altered in inherited neurometabolic conditions and that its modulation may represent an interesting therapeutic strategy for these disorders.

## Nrf2 Signaling Pathway Overview

The Nrf2, encoded by *NFE2L2*, is a basic leucine zipper transcription factor, member of the cap “n” collar proteins (Sykiotis and Bohmann, 2010) containing seven Nrf2-ECH homology (Neh) functional domains, Neh1-Neh7 (Canning et al., 2015). Nrf2 is located in the cytosol in an inactive form bound to Kelch-like ECH associated protein-1 (Keap1) through DLG and ETGE motifs present in the Neh2 domain. Keap1 also binds cullin 3 (CUL3), a member of cullin proteins that play a role in ubiquitination (Andérica-Romero et al., 2013; Ahmed et al., 2017). CUL3 activates the ubiquitination process and hence the proteasome degradation of Nrf2. This Keap1 activity explains the instability of Nrf2, which has a half-life of 15–40 min and low abundance under basal conditions (Cuadrado et al., 2019).

**FIGURE 1:**
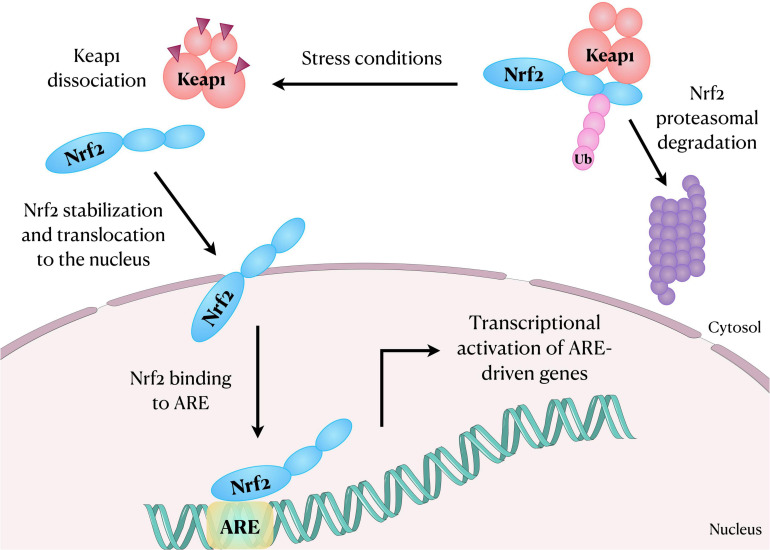
Schematic representation showing Nrf2 translocation to the nucleus after dissociation from its inhibitory protein Keap1. In the nucleus, Nrf2 binds and activates ARE in the DNA, regulating the expression of several genes, including those involved in oxidative stress responses. ARE, antioxidant response element; Keap1, Kelch-like ECH associated protein-1; Nrf2, nuclear factor erythroid-2-related factor 2; Ub, Ubiquitin.

The Nrf2 dissociates from Keap1 due to the thiol modification of Keap1 cysteine residues following oxidative stress or exposure to activators (Yamamoto et al., 2018). Nrf2 then translocates into the nucleus, heterodimerizes with musculoaponeurotic fibrosarcoma (Maf) proteins through Neh1 domain and transactivates an antioxidant response element (ARE) (Figure 1). The Neh3, Neh4, and Neh5 domains are the transactivation domain and Neh5 is fundamental for the regulation and cellular localization of Nrf2 (Li et al., 2006). It is established that Nrf2-ARE interaction regulates the expression of about 250 genes, with emphasis to genes involved in oxidative stress responses (Cuadrado et al., 2019). For instance, Nrf2 regulates the expression of glucose 6-phosphate dehydrogenase, 6-phosphogluconate dehydrogenase, malic enzyme 1, and isocitrate dehydrogenase 1 genes, which are involved in the generation of NADPH, a crucial cofactor for many redox enzymes. It also regulates the expression of heme oxygenase-1 (HO-1) and enzymes involved in the synthesis and use of reduced glutathione (GSH), such as subunits of glutamate-cysteine ligase, glutathione reductase, glutathione peroxidase (GPx) and several glutathione S-transferases (Cuadrado et al., 2019). It is also noteworthy that Nrf2 further regulates the expression of some cytochrome P450 oxidoreductases (Cuadrado et al., 2019). In this scenario, mounting evidence has suggested that the activation of Nrf2-ARE signaling pathway plays a role in the improvement of chronic inflammation involved in several autoimmune, autoinflammatory, metabolic, infectious, neurodegenerative diseases, and in cancer (Hennig et al., 2018). Moreover, the Nrf2 and the NLRP3 inflammasome are inversely correlated in regulating inflammation.

Other mechanisms independent of Keap1 for Nrf2 regulation have been proposed. The Neh6 domain of Nrf2 binds β-transducin repeat-containing protein (β-TrCP) that marks Nrf2 for ubiquitination and proteasomal degradation. The glycogen synthase kinase-3 (GSK3) phosphorylates Nrf2 in the Neh6 domain to expedite the recognition of Nrf2 by β-TrCP (Rada et al., 2011; Chowdhry et al., 2013). In addition, an alternative pathway where the p62, an autophagosome cargo protein (Liu et al., 2016), induces autophagic degradation of Keap1 and the release of Nrf2 has been also shown (Komatsu et al., 2010; Lau et al., 2010).

## Brain Metabolism and Nrf2 Signaling

The brain has a high metabolic rate and consumes about 20% of inhaled O_2_ suggesting a high-energy-demanding tissue. Although a variety of carbon sources may be oxidized, glucose is the primary fuel in the adult brain, which constitutes only about 2% of the total body weight but takes up to 20% of the body’s glucose disposal at rest (Bolaños, 2016; Belenguer et al., 2019). Neurotransmission is the process responsible for most of the neural energy expenditure, requiring a continuous control of ion gradients across the plasma membrane through primary active transporters (Bolaños, 2016; Belenguer et al., 2019). Thus, neurotransmission unavoidably increases reactive species generation in neurons due to enhanced oxidative phosphorylation (Bolaños, 2016). Furthermore, the brain is especially rich in redox transition metals, favoring the Fenton reaction and generating highly oxidant radicals, which easily react with polyunsaturated lipids, forming lipid radicals and other products. Of note, these products can react with amino acids residues, altering structural, catalytic and transport proteins. Since polyunsaturated fatty acids are abundant in the brain, their oxidation may cause damage to membranes and consequently affect neurotransmission, signal transduction, ion transport and electrical conduction (Díaz et al., 2021).

Although brain metabolism generates considerable levels of ROS, neuronal antioxidant defenses are not sufficient to cope with these species. For instance, concentrations of GSH are remarkably low in nerve cells and becomes even lower with aging (Díaz et al., 2021). Weak neuronal antioxidant defenses are also a result of the continuous protein destabilization of Nrf2, the master antioxidant transcriptional activator (Jimenez-Blasco et al., 2015). Nevertheless, Nrf2 is highly stable in astrocytes, conferring a more efficient antioxidant system to these cells compared to neurons (Bolaños, 2016). Accordingly, the levels of products from gene activation mediated by Nrf2 are higher in astrocytes than in neurons (Rosito et al., 2020). In this scenario, astrocytes play a key role in providing antioxidant support to nearby neurons by releasing GSH precursors, which are taken up by neurons and used to synthesize their own GSH *de novo* (Baxter and Hardingham, 2016; Rosito et al., 2020). Moreover, it has been proposed that endogenous and exogenous modulation of Nrf2 expression could increase the expression of specific genes resulting in neuroprotection and neurogenesis, since Nrf2 plays an important role in neural stem cells, maintaining the homeostasis of neural tissue and supporting neurogenesis in physiological and pathological conditions (Qiu et al., 2020; Kahroba et al., 2021).

Since Nrf2 signaling pathway plays an important role in the redox regulatory system between astrocytes and neurons and oxidative stress is commonly observed as a pathomechanism in many IMDs (Mc Guire et al., 2009; Ribas et al., 2014; Grings et al., 2020; Wajner et al., 2020; Ravi et al., 2021), it is conceivable that alterations in Nrf2 expression are involved in the pathophysiology of neurometabolic disorders, representing a promising target for the development of novel therapies that may improve the quality of life of patients.

## Nrf2 Signaling Disruption in Inherited Metabolic Disorders

### X-Linked Adrenoleukodystrophy

X-linked adrenoleukodystrophy (X-ALD) (OMIM 300100) is a complex molecule defect, caused by mutations in the ABCD1 (ATP-binding cassette, subfamily D, member 1) gene, which encodes the peroxisomal ABC half-transporter ALD protein. While it is estimated to affect approximately 1 in 42,000 males, it has been also reported that 1 in 28,000 females are estimated to be heterozygous for an ABCD1 mutation. The deficient ALD protein leads to abnormal breakdown of very long-chain fatty acids (VLCFA), a process that predominantly affects adrenal and nervous system tissues. Thus, X-ALD is biochemically characterized by elevated levels of VLCFA, especially C26:0, in tissues and plasma and constitute a pathognomonic biomarker for diagnosis (Engelen et al., 2012; Kemper et al., 2017).

X-linked adrenoleukodystrophy has a broad phenotype and there is no genotype-phenotype correlation, even within families (Wiesinger et al., 2015), suggesting the influence of modifier genes or environmental factors (Berger et al., 1994; Turk et al., 2017). Two main forms of the disease have been described (Engelen et al., 2012). The most severe form, childhood cerebral ALD, affects between 31 and 57% of hemizygous males and is mostly present in boys between 5 and 10 years (35–40% of the cases), who present a strong inflammatory demyelinating reaction in CNS white matter. Patients present with behavioral changes including memory impairment and emotional instability, followed by progressive deterioration of the vision, hearing, and motor function. Adrenal dysfunction or gonadal insufficiency may be also seen, besides the CNS symptoms (Engelen et al., 2012, 2014). The other form is called adrenomyeloneuropathy (AMN) and occurs in 60% of the cases, affecting adult men and heterozygous women over the age of 40 (Engelen et al., 2014). AMN is characterized by peripheral neuropathy and distal axonopathy involving the corticospinal tract of the spinal cord. In addition to myeloneuropathy, around 80% of all male X-ALD patients develop adrenocortical insufficiency (Dubey et al., 2005). Symptomatology includes progressive stiffness and weakness of the legs, sphincter disturbances, sexual dysfunction, a baldness pattern and increased skin pigmentation, clinical signs that are usually progressive over decades (Li et al., 2019).

To our knowledge, so far only one study has verified alterations in Nrf2 pathway in X-ALD (Ranea-Robles et al., 2018). Reduced Nrf2 protein levels were shown in spinal cord of *Abcd1*^–^ mice (AMN mice model) (Pujol et al., 2002), along with decreased mRNA levels of its target genes *Hmox1* (heme oxygenase-1, HO-1), *Nqo1* (NADH quinone oxidoreductase 1, NQO1) and *Gsta3* (glutathione S-transferase alpha-3) (Ranea-Robles et al., 2018). It was seen that these alterations were mediated by a dysregulation in AKT/GSK3β/Nrf2 axis with a defective AKT phosphorylation and a consequent activation of GSK3β. n addition, when patients’ fibroblasts were exposed to both C26:0, the main VLCFA accumulated in patients, and oligomycin, an ATP synthase inhibitor that induces ROS generation, there was no activation of Nrf2-dependent responses due to abnormal GSK3β activation (Ranea-Robles et al., 2018). Importantly, it was demonstrated that the addition of dimethyl fumarate (DMF), a classical activator of Nrf2, to the deficient fibroblasts reactivated Nrf2 pathway (Table 1). Moreover, feeding *Abcd1*^–^ mice with DMF-containing chow rescued Nrf2 pathway, improved antioxidant system, bioenergetics and mitochondrial biogenesis, and prevented inflammation in the spinal cord of these animals (Ranea-Robles et al., 2018). Further data on DMF beneficial effects were shown in *Abcd1*^–^/*Abcd2*^–/–^ mice, a model of X-ALD with development of a more severe and earlier onset axonopathy (Launay et al., 2015). When these mice were fed DMF, they presented a reversal of astrocytosis, microgliosis, and axonal and myelin degeneration in their spinal cord. Locomotor performance was also improved by DMF, indicating that this compound ameliorated the neuropathology of X-ALD (Ranea-Robles et al., 2018).

**TABLE 1:** Summary of Nrf2 modulators evaluated in cellular and animal models, as well as in samples from patients affected by inherited metabolic disorders (IMD).

Compound	IMD	Effects	Active/completed clinical trials	References
Dimethyl fumarate (DMF)	X-linked Adrenoleukodystrophy (X-ALD)	Promotes Nrf2 nuclear translocation by interacting with Keap1 cysteine residues. Increases Nrf2 mRNA levels.	–	Ranea-Robles et al., 2018
	Friedreich’s ataxia (FRDA)			Hayashi and Cortopassi, 2016; Jasoliya et al., 2019; Petrillo et al., 2019
Sulforaphane (SFN)	Friedreich’s ataxia (FRDA)	Promotes Nrf2 nuclear translocation by interacting with Keap1 cysteine residues. Increases Nrf2 mRNA levels.	–	Abeti et al., 2015; Petrillo et al., 2017, 2019; La Rosa et al., 2019, 2021
TBE-31	Friedreich’s ataxia (FRDA)	Promotes Nrf2 nuclear translocation by interacting with Keap1 cysteine residues. Increases Nrf2 mRNA levels.	–	Abeti et al., 2015
RTA 408 (Omaveloxolone; Omav)	Friedreich’s ataxia (FRDA)	Promotes Nrf2 nuclear translocation by interacting with Keap1 cysteine residues. Increases Nrf2 mRNA levels.	Phase II clinical trial (ClinicalTrials.gov Identifier: NCT02255435; MOXIe)	Abeti et al., 2018; Lynch et al., 2018, 2021; Petrillo et al., 2019
N-acetylcysteine (NAC)	Friedreich’s ataxia (FRDA)	Antioxidant that induces Nrf2 expression.	–	Petrillo et al., 2019
EPI-743	Friedreich’s ataxia (FRDA)	Promotes Nrf2 nuclear translocation. Increase Nrf2 mRNA levels.	–	La Rosa et al., 2019, 2021; Petrillo et al., 2019
Idebenone	Friedreich’s ataxia (FRDA)	CoQ10 analog that induces Nrf2 expression.	–	Petrillo et al., 2019
Dyclonine	Friedreich’s ataxia (FRDA)	Activates the ARE/Nrf2 pathway, by enhanced binding of Nrf2 to ARE sites.	–	Sahdeo et al., 2014

### Hyperhomocysteinemia

Hyperhomocysteinemia (HHcy) is a condition characterized by high plasma levels of the amino acid homocysteine (Hcy) (greater than 15 μmol/L) (Kim et al., 2018), a non-protein sulfur amino acid originated from the essential amino acid methionine. HHcy is an independent risk factor for the development of various serious medical conditions, such as neurodegenerative, cardiovascular, cerebrovascular, and thromboembolic diseases (Son and Lewis, 2021).

Homocysteine is synthesized by the demethylation of methionine via formation of two intermediate compounds, S-adenosylmethionine (SAM) and S-adenosylhomocysteine (SAH). Methionine is first converted to SAM through the catalytic action of methionine adenosyltransferase. Different methyltransferases remove the methyl group from SAM generating SAH, which is then converted into Hcy and adenosine by SAH hydrolase The formation of Hcy from methionine is the only pathway of Hcy biosynthesis in humans (Barić et al., 2017; Cordaro et al., 2021).

In turn, Hcy may be converted to methionine and cysteine by the action of different enzymes and a combination of B vitamins (B12, B6, and folate). Specifically, Hcy is converted to methionine by a process known as remethylation, that can occur by a folate-dependent or -independent pathway. 5-Methyltetrahydrofolate (5-MTHF) is the active folate derivative and the main circulating form of folate in plasma. In the folate-dependent pathway, 5-MTHF supplies the methyl group used by the vitamin B12-dependent methionine synthase to remethylate Hcy and produce methionine and tetrahydrofolate (THF). THF is then converted to 5,10-methylenetetrahydrofolate (5,10-MeTHF) in the presence of serine and vitamin B6 by the enzyme serine hydroxymethyltransferase e. 5,10-MeTHF is reduced to 5-MTHF by 5,10-methylenetetrahydrofolate reductase (MTHFR) with flavin adenine dinucleotide as cofactor, being available for the remethylation of a second molecule of Hcy. The folate-dependent remethylation pathway is present in nearly all cells. Additionally, in liver and kidney, remethylation also occurs by the folate-independent pathway in which methyl groups are donated by betaine in a reaction catalyzed by the enzyme betaine-homocysteine methyltransferase (Barić et al., 2017; Cordaro et al., 2021).

On the other hand, Hcy may be irreversibly converted to cysteine through the transsulfuration pathway. In the first step, the enzyme cystathionine β-synthase (CBS) catalyzes the condensation of Hcy and serine to form cystathionine using pyridoxal phosphate (PLP or vitamin B6) as a cofactor. Cystathionine is further metabolized by cystathionine γ-lyase using PLP, to produce cysteine (Barić et al., 2017; Cordaro et al., 2021).

Elevations in Hcy levels may be caused by deficiency in any of the components of these reactions. Owing to the crucial role of vitamins in Hcy metabolism, any causes of vitamin B12, B6, and folate deficiency (i.e., alcohol use, proton pump inhibitors) can induce elevation of Hcy levels (Son and Lewis, 2021). Classic homocystinuria is an autosomal recessive disorder caused by mutations in *CBS* gene on chromosome 21q22.3 leading to deficient activity of CBS (OMIM 236200). It belongs to the category of the disorders of small molecules. The clinical features usually manifest in the first or second decade of life and include myopia, ectopia lentis, intellectual disability, skeletal anomalies and thromboembolic events. Biochemical features include increased urinary excretion of Hcy and methionine. There are two main phenotypes of the classic disorder: a milder pyridoxine (vitamin B6)-responsive form, and a more severe pyridoxine-non-responsive form (Reish et al., 1995; Testai and Gorelick, 2010). Another common cause of HHcy is the deficient enzyme activity of MTHFR caused by mutations in the gene *MTHFR*, on chromosome 1p36.22, a common inherited disorder of folate metabolism (OMIM 236250). The individuals diagnosed with this disorder present with a phenotypic spectrum that ranges from severe neurologic deterioration and early death to asymptomatic adults (Goyette et al., 1995; Strauss et al., 2007).

Data have shown the involvement of Nrf2 signaling pathway in HHcy. Alterations in this pathway as well as oxidative stress were verified in prefrontal cortex and amygdala of Wistar rats that received chronic Hcy administration (a model of mild HHcy) (Dos Santos et al., 2019). A marked increase of Nrf2 protein content was seen in nucleus of rat amygdala, whereas no alterations were observed in the cytosol of amygdala as well as in nucleus and cytosol of pre-frontal cortex (Dos Santos et al., 2019). Furthermore, the increased levels of Nrf2 in the amygdala were seen to occur concomitantly with augmented activities of superoxide dismutase (SOD), GPx, and catalase (CAT), suggesting that the translocation of this transcription factor to nucleus correlated with higher expression of antioxidant defenses. Increased nitrite levels and DNA damage, probably provoked by higher levels of reactive oxygen and nitrogen species, were also verified in amygdala (Dos Santos et al., 2019). In addition, chronic HHcy also compromised the mitochondrial respiratory chain with a consequent reduction of ATP levels in the amygdala, effects that may have also contributed to the induction of Nrf2 translocation (Dos Santos et al., 2019).

Another study investigated the effects of chronic Hcy administration, as well as the influence of hydrogen sulfide treatment in adult Sprague Dawley rats (Kumar and Sandhir, 2018). Of note, hydrogen sulfide is suggested to mediate S-sulfhydration of Keap1, thus leading to Nrf2 activation (Xie et al., 2016; Kumar and Sandhir, 2018). Marked alterations in oxidative stress parameters were induced by Hcy in rat cerebral cortex and hippocampus, including lipid peroxidation, increased levels of ROS, protein carbonyls and 4-hydroxynonenal-modified proteins, as well as decreased GSH/GSSG ratio and SOD, GPx, glutathione reductase, and glutathione-S-transferase activities (Kumar and Sandhir, 2018). All these effects were prevented by hydrogen sulfide (Kumar and Sandhir, 2018). The same study also evidenced a decrease of cytosolic protein levels and mRNA expression of Nrf2 in cerebral cortex of Hcy-treated animals, without changes in hippocampus. However, nuclear Nrf2 content was decreased in both cerebral cortex and hippocampus. Hydrogen sulfide supplementation significantly increased Nrf2 protein and mRNA levels in both brain structures. Overall, these findings indicate that disturbances in Nrf2 signaling pathway caused by Hcy possibly contribute to the alterations in redox homeostasis.

The studies aforementioned reported different results regarding the alterations on Nrf2 levels caused by Hcy, though the same dose of Hcy was used in both studies (0.03 μmol/g of body weight). While the first report showed that Hcy increased Nrf2 protein levels (Dos Santos et al., 2019), the second demonstrated that Hcy treatment decreased mRNA and protein levels of Nrf2 (Kumar and Sandhir, 2018). Nevertheless, these apparent controversial results may be explained by differences in the rat species used in both studies, as well as the age of the animals.

In addition, there is mounting evidence that Hcy also alters Nrf2 signaling in other rodent tissues. In this regard, a decrease of Nrf2 levels was observed in lens epithelial cells exposed to Hcy (experimental model to study cataracts) (Elanchezhian et al., 2012; Yang et al., 2015). On the other hand, another report showed increased ROS and Nrf2 levels in retina of hyperhomocysteinemic mice (Mohamed et al., 2017).

### Glutaric Acidemia Type I

Glutaric acidemia type I (GA I) (OMIM 231670) is an autosomal recessive disorder caused by mutations in the gene *GCDH*, located on the short arm of the chromosome 19, leading to deficient activity of the mitochondrial enzyme glutaryl-CoA dehydrogenase (GCDH). This cerebral organic aciduria is categorized as a disorder of small molecules The enzyme GCDH participates in the metabolic pathway of the amino acids lysine (Lys), tryptophan and hydroxylysine, converting glutaryl-CoA to crotonyl-CoA in a two-step reaction (Larson and Goodman, 2019). The biochemical profile of GA I patients includes the accumulation of the metabolites glutarylcarnitine, glutaric acid (GA), and 3-hydroxygluric acid (3HGA) in all tissues of the patients, but predominantly within the brain (Kölker et al., 2006). The predicted worldwide frequency of GA I ranges from 1:30,000 to 1:100,000 newborns, being one of the most prevalent organic acidurias (Wajner, 2019). Affected patients have encephalopathic crises manifested by convulsions during the first 3 years of life, which are normally provoked by catabolic events, such as fever, infection or prolonged fasting. These events usually cause irreversible striatum degeneration, resulting in dystonia, dyskinesia, muscle stiffness and general developmental deterioration (Strauss and Morton, 2003). Gliosis and neuronal loss especially in the basal ganglia are also commonly observed (Larson and Goodman, 2019; Wajner, 2019).

A knockout model of GA I (*Gcdh*^–/–^) was developed in mice by replacing exons 1–7 of the *GCDH* gene with the *nlacF* and *NEO* genes (Koeller et al., 2002). Exposing *Gcdh*^–/–^ animals to high protein or Lys intake resulted in elevated serum and brain GA and 3HGA accumulation, as well as neuronal loss, myelin disruption and gliosis mostly in the striatum and deep cortex (Zinnanti et al., 2006, 2007). Therefore, the *Gcdh*^–/–^ mouse model exposed to Lys overload has been preferentially used as an appropriate GA I animal model to study the neuropathology of this disorder (Wajner et al., 2019). Different approaches were used to achieve high Lys levels in these animals, including Lys intraperitoneal or intracerebral injections, as well as chow supplemented with Lys (Zinnanti et al., 2006; Wajner et al., 2019).

Oxidative stress and neuroinflammation have been postulated to be involved in the pathogenesis of GA I, as observed by many studies using GA I animal models and cells from patients (Wajner, 2019; Wajner et al., 2019; Guerreiro et al., 2021), as well as of many other neurologic diseases (Freeman and Ting, 2016). As for the role of oxidative stress and Nrf2 pathway disruption in the pathophysiology of GA I, a study showed that the administration of quinolinic acid (QA), a metabolite synthesized by the kynurenine pathway during inflammatory process, induced oxidative stress in young *Gcdh*^–/–^ mice fed a high Lys chow (Seminotti et al., 2016). In detail, QA induced lipid and protein oxidative damage, disturbed antioxidant defenses and increased reactive species production in striatum of *Gcdh*^–/–^ mice fed a high Lys chow (Seminotti et al., 2016). All these changes occurred in parallel with increased levels of the transcription factors Nrf2 and NF-κB in the nucleus, as well as augmented Erk1/2 phosphorylation and Akt levels. Importantly, both ERK and Akt are kinases that participate in various signaling pathways, including Nrf2 translocation (Ishii and Warabi, 2019). Moreover, Keap1 and IκBα, the inhibitory proteins of Nrf2 and NF-κB, respectively, were seen to be decreased in the cytosol *Gcdh*^–/–^ mice (Seminotti et al., 2016).

Another work showed that a single intrastriatal administration of Lys to young *Gcdh*^–/–^ mice augmented Nrf2 protein expression in the nucleus of striatum (Amaral et al., 2019). It was also found that NF-κB expression was increased whereas HO-1 content was decreased in the striatum of these animals (Amaral et al., 2019).

Taken together, these findings demonstrated that Nrf2 signaling and redox homeostasis are altered in brain of *Gcdh*^–/–^ mice submitted to Lys overload, effects that are induced at least partially by increased brain concentrations of GA and 3HGA derived from high Lys levels (Wajner, 2019).

### Friedreich’s Ataxia

Friedreich’s ataxia (FRDA) (OMIM 229300) is the most common autosomal recessive inherited ataxia, with an estimated prevalence of 1:50,000 (Dürr et al., 1996). The first symptoms usually appear between 10 and 15 years of age due to progressive degeneration of large dorsal root ganglion (DRG) cells, dorsal spinocerebellar, and corticospinal tracts, as well as dentate nucleus of the cerebellum and other nuclei (Dürr et al., 1996; Koeppen and Mazurkiewicz, 2013; Patel et al., 2016). This results in neurological manifestations that include cerebellar and sensory ataxia, dysarthria, lack of deep tendon reflexes, pyramidal weakness of the legs, optic atrophy and visual and hearing impairment. Systemic presentations, such as hypertrophic cardiomyopathy, diabetes mellitus, kyphoscoliosis, and pes cavus, may be also observed (Kipps et al., 2009; Reetz et al., 2018; Hanson et al., 2019; Pandolfo, 2019). Patients usually lose the ability to ambulate around their mid-20 s (Campuzano et al., 1996; Lynch et al., 2021). Less frequent late onset (after 25 years) and very late-onset (after 40 years) FRDA variants commonly present with milder phenotypes and absence of systemic symptoms (Koeppen et al., 2011). Currently, there is no approved therapy for this disorder.

In most cases, FRDA occurs as a consequence of homozygous expanded guanosine-adenosine-adenosine (GAA) repeats in the first intron of the *FXN* gene, which usually contains up to 40 GAA triplets and may increase to over 1,700 in disease-associated alleles. The number of repeats is associated with earlier onset and disease severity (Campuzano et al., 1996; Dürr et al., 1996). As a result of the GAA expansion, the *FXN* gene is partially silenced and lower levels of the gene-encoded protein frataxin are expressed (Groh et al., 2014). The rare non-expansion mutations, on the other hand, may lead to the synthesis of partial or non-functional protein (Correia et al., 2008). Frataxin is a mitochondrial protein involved in cellular iron homeostasis and functions as a chaperone during iron-sulfur cluster and heme synthesis by incorporating iron to their precursors (Yoon and Cowan, 2004). The deficiency of frataxin has been associated with reduced activity of mitochondrial respiratory chain complexes, lower ATP production and decreased mitochondrial content, as well as iron accumulation and oxidative stress (Lodi et al., 1999; Bradley et al., 2000; Schulz et al., 2000; Jasoliya et al., 2017; Lin et al., 2017; Doni et al., 2021). Even though it was not previously classified (Saudubray and Garcia-Cazorla, 2018a), since the deficient protein leads to mitochondrial iron overload and consequent defective energy supply (Yoon and Cowan, 2004), FRDA might be categorized either in the group of small molecule defects or energy-related disorders. However, it remains to be stablished.

Nuclear factor erythroid-2-related factor 2 signaling pathway has been shown to be disrupted in patients’ cells and different FRDA models, rendering the cells more susceptible to oxidative damage due to decreased antioxidant dxsefenses (Wong et al., 1999; Chantrel-Groussard et al., 2001; Sturm et al., 2005; Al-Mahdawi et al., 2006). Initial evidence of decreased Nrf2 signaling in FRDA was demonstrated by Paupe et al. (2009). Upon treatment of FRDA fibroblasts with oligomycin and tert-butylhydroquinone (tBHQ) to induce oxidative stress, deficient cells did not present increased nuclear translocation of Nrf2, in contrast to normal cells (without deficiency). In addition, while tBHQ and oligomycin treatment increased mRNA levels of antioxidant enzymes regulated by Nrf2 in normal fibroblasts, FRDA fibroblasts failed to induce the expression of these proteins. Similar findings were observed in neuroblastoma-derived cell lines (SKNAS) challenged with tBHQ and oligomycin (Paupe et al., 2009). Furthermore, frataxin-deficient motor neuron-like cells (NSC34) exposed to GSSG also failed to induce nuclear translocation of Nrf2 (D’Oria et al., 2013).

Another study did not observe altered Nrf2 nuclear translocation in frataxin-deficient HeLa cells and DRG neurons (ND7/23), as well as in patient’s lymphoblasts when treated with tBHQ. On the other hand, a reduction in Nrf2 content was seen in these cells, as well as in frataxin-deficient fibroblasts and Schwann cells (T265 cell line). In line with this, protein content and activity of thioredoxin reductase 1 (TxnRD1) were also decreased in some of these cells. Furthermore, transcripts and protein levels of Nrf2 were found reduced in DGR tissue and cerebellum of YG8R mice (FRDA model), which express expanded mutant alleles of human frataxin. This reduction was accompanied by lower GSH levels and protein content of HO-1, NQO1, and SOD2 in DRG. Moreover, in cerebellum and DRG Nrf2 expression was correlated with frataxin expression, which was further correlated with Nrf2-regulated genes in DRG (Shan et al., 2013). NSC34 neurons also presented a decrease in basal Nrf2 transcript and protein content along with reduced SOD and GST levels (D’Oria et al., 2013). Downregulation of Nrf2 pathway was further observed in peripheral blood cells of FRDA patients (Haugen et al., 2010).

The mechanisms underlying the impairment of Nrf2 signaling are not fully understood. Nevertheless, it was observed that under basal conditions the Nrf2 transcription factor is abnormally located in FRDA fibroblasts, which could be involved in the dysfunctional Nrf2 activation. In detail, while in normal fibroblasts Keap1 and Nrf2 were associated to actin stress fibers, in patient cells Keap1 and Nrf2 were not bound to these fibers, which were atypically distributed in the cell periphery (Paupe et al., 2009). This is in agreement with previous studies that showed disorganized actin structure, as well as increased actin glutathionylation in FRDA fibroblasts, an alteration that may disturb cytoskeleton stabilization (Pastore et al., 2003). Another study performed in a mouse conditional frataxin knockout model in heart and skeletal muscle showed that cardiac frataxin deficiency resulted in enhanced Keap1 expression and GSK3β-induced activation of a nuclear export machinery, leading to reduced cytosolic and nuclear Nrf2, respectively. Interestingly, although a decrease of mRNA levels of Nrf2 downstream antioxidant genes was observed, the correspondent proteins presented increased or unaltered content. No changes in Nrf2 signaling were found in the skeletal muscle of conditional knockout mice (Anzovino et al., 2017). Consistent with this study, the content of Keap1 was augmented, whereas DJ-1, a protein that stabilizes Nrf2 by preventing its association with Keap1, was reduced in FRDA fibroblasts (Petrillo et al., 2019).

Other studies explored Nrf2 signaling as a potential therapeutical target for FRDA by evaluating the effects Nrf2-activating compounds in FRDA models and patients. Among these compounds, sulforaphane (SFN), DMF, TBE-31 and RTA 408 (Omaveloxolone; Omav) are known to directly activate Nrf2 due to their interaction with Keap1 cysteine residues, which inhibits Nrf2 ubiquitination and degradation (Takaya et al., 2012; Kostov et al., 2015; Shekh-Ahmad et al., 2018). Other compounds, such as *N*-acetylcysteine (NAC), EPI-743, idebenone and dyclonine, also induced Nrf2 expression in different FRDA models (Table 1).

Additionally, it was observed that fibroblasts from the FRDA mice models YG8R and KIKO presented increased susceptibility to hydrogen peroxide and that treatment with SFN and TBE-31 prevented the hydrogen peroxide-induced decrease of cell viability. Of note, KIKO mouse model is characterized by knock-in-expanded GAA repeat on one allele (230 GAAs) and a knockout of FXN on the other allele, causing moderate overall deficiency of frataxin early in life. Lipid peroxidation and mitochondrial membrane depolarization were also ameliorated in these fibroblasts by both SFN and TBE-31 (Abeti et al., 2015). Moreover, treatment of FRDA patient’s fibroblasts with SFN induced higher mRNA levels of Nrf2 and downstream genes (Petrillo et al., 2017). Similarly, in frataxin-deficient motor neurons, SFN and DMF augmented Nrf2 mRNA and protein levels, in addition to protein content of Nrf2 downstream genes. A decrease in GSSG/GSH ratio and axonal regrowth, with a reorganization and increased number of neurites, were also observed with SFN and DMF treatment (Petrillo et al., 2017). Noteworthy, La Rosa et al. (2019) found that such defects in the Nrf2 pathway may occur at early stages of neurogenesis, and treatment with SFN and EPI-743 showed beneficial effects in KIKO mouse embryonic cortex neural stem cells. Besides enhancing mRNA and protein levels of Nrf2 and its downstream target genes, SFN and EPI-743 decreased ROS overload and, importantly, reestablished the cellular differentiation program in these cortex neural stem cells, increasing the number and the length of neurites.

Sulforaphane and EPI-743 were further shown to rescue alterations associated with ferroptosis in FRDA fibroblasts by promoting Nrf2 nuclear translocation, decreasing lipid peroxides and glutathionylated proteins levels, and restoring the mitochondrial morphology from small and fragmented organelles to tubular networks (La Rosa et al., 2021). Ferroptosis-related alterations, such as increased content of the lipid peroxidation marker 4-hydroxynonenal and reduced expression of Nrf2 and some of its target genes, were also ameliorated in leukocytes of FRDA patients treated with idebenone (La Rosa et al., 2021).

In contrast to SFN and DMF that possess effects on a wide range of proteins, cyanoenone triterpenoids, such as Omav, are more potent and target specifically Nrf2 activation (Satoh and Lipton, 2017; Shekh-Ahmad et al., 2018). In this regard, it was observed that treatment of cerebellar granule neurons from KIKO and YG8R mice with Omav restored the activity of respiratory chain complex I, which is highly dependent on iron-sulfur clusters. Similar findings were verified in FRDA patient’s fibroblasts. Omav reverted increased lipid peroxidation and mitochondrial ROS levels, as well as decreased GSH content under basal conditions in FRDA fibroblasts. Upon challenge with hydrogen peroxide, Omav further protected fibroblasts from mitochondrial membrane potential dissipation and cell death (Abeti et al., 2018). Noteworthy, Omav is currently in Phase II clinical trial in FRDA patients (ClinicalTrials.gov Identifier: NCT02255435; MOXIe), showing improvement in the neurological dysfunction and general safety (Lynch et al., 2018, 2021).

Other investigations evaluated the effects of these Nrf2-activating compounds on the expression of frataxin, the primary defect in FRDA, in parallel to the alterations seen on Nrf2 pathway. In this regard, a study showed that dyclonine, an oral anesthetic that confers topical anesthesia to mucous membranes, induced frataxin expression in FRDA lymphoblasts and cerebellum of YG8R and KIKO mice, as well as in buccal cells of FRDA patients (Sahdeo et al., 2014). Dyclonine was found to activate the ARE/Nrf2 pathway in YG8R mouse cerebellum. Interestingly, it was verified that ARE sites are present in the *FXN* gene, and dyclonine-treated FRDA lymphoblasts had enhanced binding of Nrf2 to these sites in the *FXN* and *Hmox1* genes (Sahdeo et al., 2014).

Another work compared the effects of SFN, DMF, Omav, idebenone, EPI-743 and NAC (redox-active compound) on Nrf2 pathway activation and frataxin expression in FRDA fibroblasts (Petrillo et al., 2019). Although these compounds elicited differential effects on Nrf2 target genes and GSH levels, all of them augmented previously reduced Nrf2 transcripts in these cells. Moreover, SFN, DMF, NAC, and EPI-743 increased frataxin transcripts (Petrillo et al., 2019). Further data demonstrated increased levels of frataxin mRNA levels in FRDA lymphoblasts and fibroblasts, and elevated frataxin protein content in cerebellum of YG8R and KIKO mice treated with DMF (Hayashi and Cortopassi, 2016; Jasoliya et al., 2019). It was also found in FRDA lymphoblasts that DMF stimulated frataxin expression (Jasoliya et al., 2019).

Finally, a recent study analyzed the GSH system and Nrf2 signaling in the modulation of the phenotype of a family with a proband affected by late-onset FRDA and his asymptomatic mother and younger sister, that also presented decreased frataxin levels (Petrillo et al., 2021). Surprisingly, the affected proband presented high levels of GSH and low levels of GSSG, while the asymptomatic mother and sister had low GSH and increased GSSG in leukocytes. Similar findings were observed in fibroblasts. Additionally, the unaffected mother presented higher mRNA levels of GCL, which participates in GSH synthesis, and of GR in leukocytes and fibroblasts, probably as a compensatory mechanism. The affected proband and the unaffected sister only presented increased transcripts of GR. Interestingly, the mother and the younger sister presented augmented Nrf2 expression in leukocytes and fibroblasts, whereas induction of Nrf2 was not found in fibroblasts of the proband. However, Nrf2 levels were increased in leukocytes of the proband, which could be a consequence of idebenone treatment, as reported previously (Petrillo et al., 2019, 2021; La Rosa et al., 2021).

In summary, these data not only reinforce the involvement of Nrf2 signaling disruption in the neuropathophysiology of FRDA but also show that this signaling pathway could be a promising therapeutical target in this disorder. Additional investigations are also needed to better clarify the initial cause of dysfunctional Nrf2 signaling in different tissues affected in FRDA, especially in neuronal models.

## Conclusion and Future Directions

Inherited metabolic disorders are caused by abnormal functioning or reduced levels of specific proteins related to metabolic pathways. Disturbances in these metabolic pathways result in a spectrum of clinical findings affecting multiple organs, predominantly the nervous system (Saudubray and Garcia-Cazorla, 2018a). Regarding the mechanisms involved in the pathophysiology of these disorders, mounting evidence has shown that oxidative stress often plays a key role in IMDs clinically characterized by neurological dysfunction (Ristoff and Larsson, 2002; Mc Guire et al., 2009; Leipnitz et al., 2015; Zubarioglu et al., 2017; Cansever et al., 2019; Wajner, 2019; Wyse et al., 2019, 2021; Grings et al., 2020; Ikawa et al., 2021). Therefore, it is not surprising that Nrf2 signaling disruption is also an important pathomechanism, although only few studies have investigated this transcription factor in IMDs.

**FIGURE 2:**
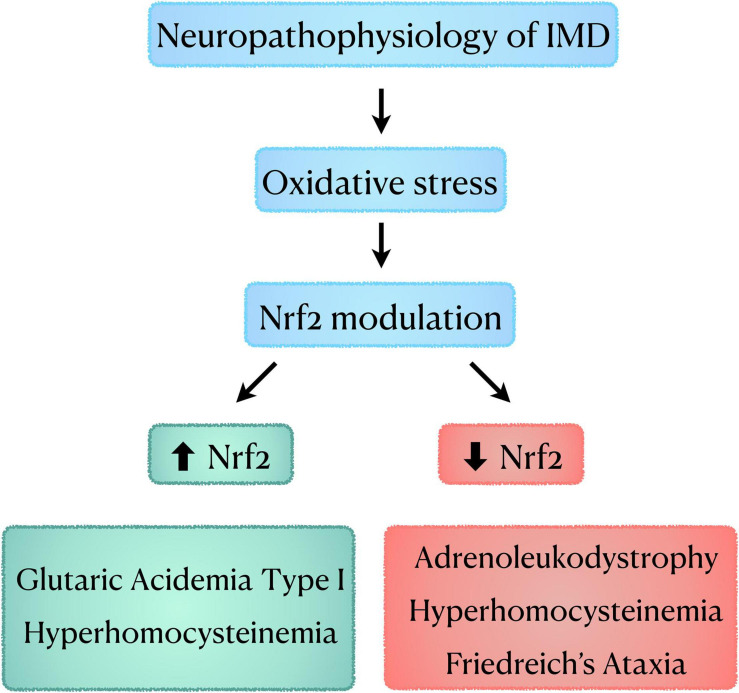
Schematic diagram showing the role of oxidative stress and Nrf2 modulation in the neuropathophysiology of IMD. IMD, Inherited Metabolic Disorders; Nrf2, nuclear factor erythroid-2-related factor 2.

We reviewed here data demonstrating that Nrf2 factor is differentially modulated not only among different IMDs (e.g., GA I × adrenoleukodystrophy) but also in different animal models of the same disease (HHcy), as well as in patient cells (FRDA) (Figure 2). When analyzing the data for HHcy, it seems that Nrf2 response was dependent on the rodent species used for each disease model and age of animals. Thus, if we extrapolate these data for the human condition, it may be speculated that the age of patients influences Nrf2 response. This is in line with data showing that Nrf2 response varies with age even in normal individuals (Schmidlin et al., 2019). Furthermore, in the studies focusing on the investigation of Nrf2 activators in FRDA models, it was seen that different compounds induced variable sets of Nrf2 downstream genes and had different effects on frataxin levels, which were dependent on the cell type (model) evaluated. This implies the existence of drug and cellular-specific transcriptional regulatory mechanisms for these genes. Another important factor that may influence Nrf2 alterations seen in IMDs is whether the patients have a residual activity of the deficient enzyme, or it is null. Noteworthy, the degree of enzymatic activity often correlates with the disease phenotype (e.g., higher enzyme activity usually causes milder phenotypes) (Gieselmann, 2005; Oliveira and Ferreira, 2019; Grünert et al., 2021; Zanfardino et al., 2021). As for FRDA, it should be highlighted that the content of frataxin may influence Nrf2 activation. Indeed, a correlation between frataxin and Nrf2 levels, as well as between frataxin levels and Nrf2 downstream genes has been verified in animal studies (Shan et al., 2013).

More studies with different models (cultured cells, patients’ cells, biological fluids and animal models) are necessary in order to determine how Nrf2 response is modulated in IMDs, and whether it is dependent on different factors (age, mutation, phenotype, etc.). It is also expected that future studies may encourage the investigation of pharmacological therapies aiming at Nrf2 modulation and consequent amelioration of oxidative stress state in these disorders.

## Author Contributions

BS and MG searched for the literature material. BS designed the figures. BS, MG, PT, GL, and LS wrote and revised the manuscript. BS and GL designed the outline. LS provided the financial support. All authors approved the last version of the manuscript.

## Conflict of Interest

The authors declare that the research was conducted in the absence of any commercial or financial relationships that could be construed as a potential conflict of interest.

## Publisher’s Note

All claims expressed in this article are solely those of the authors and do not necessarily represent those of their affiliated organizations, or those of the publisher, the editors and the reviewers. Any product that may be evaluated in this article, or claim that may be made by its manufacturer, is not guaranteed or endorsed by the publisher.
